# Effect of Graphene Oxide-Modified CaAl-Layered Double Hydroxides on the Carbon Dioxide Permeation Properties of Fluoroelastomers

**DOI:** 10.3390/polym15204151

**Published:** 2023-10-19

**Authors:** Chuanbo Cong, Daigang Peng, Qingkun Liu, Mingyang Yuan, Xiaoyu Meng, Qiong Zhou

**Affiliations:** New Energy and Material College, China University of Petroleum, Beijing 102249, China; pengdg_123@163.com (D.P.); liuqingkun1992@163.com (Q.L.); yuanmy.apply@gmail.com (M.Y.); xymeng800418@sohu.com (X.M.); zhouqiong_cn@163.com (Q.Z.)

**Keywords:** LDH, graphene oxide, permeability, carbon dioxide

## Abstract

This work aimed to investigate the CO_2_ gas barrier and mechanical properties of fluorine rubber nanocomposites filled with Ca/Al layered hydroxide (graphene oxide [GO]/LDH-Ca_2_Al) modified by GO. GO/LDH-Ca_2_Al nanocomposite fillers were prepared by depositing Ca/Al layered hydroxide (LDH-Ca_2_Al) into the surface of alkalized GO (Al-GO). The prepared GO/LDH-Ca_2_Al nanocomposite fillers and complexes were characterized by Fourier infrared spectroscopy (FTIR), X-ray diffraction (XRD), scanning electron microscopy (SEM), and transmission electron microscopy (TEM) for structural and micromorphological characterization. The results showed that GO/LDH-Ca_2_Al was successfully prepared with strong interactions between Al-GO and LDH, and the compatibility of GO/LDH-Ca_2_Al nanocomposite fillers with the polymer was significantly improved compared with that of LDH-Ca_2_Al. Consequently, both the fracture strength (*σ_b_*) and strain (*ε_b_*) of GO/LDH-Ca_2_Al nanocomplexes remarkably increased, and they exhibited excellent mechanical properties. Differential scanning calorimetry and thermogravimetric analysis were used to characterize the thermal stability of GO/LDH-Ca_2_Al nanocomposite fillers, and GO/LDH-Ca_2_Al nanocomposite fillers have better thermal stability than LDH-Ca_2_Al. The reaction products (S-LDH-Ca_2_Al and S-GO-Ca_2_Al) of LDH-Ca_2_Al and GO/LDH-Ca_2_Al with CO_2_ were characterized using XRD and TGA, respectively, and the results show that LDH-Ca_2_Al reacts readily and chemically with CO_2_, resulting in a lower diffusion coefficient of CO_2_ in the LDH-Ca_2_Al nanocomplexes than that of the GO/LDH-Ca_2_Al nanocomplexes and leading to the destruction of the laminar structure of LDH-Ca_2_Al, while GO/LDH-Ca_2_Al has better CO_2_ resistance stability. GO/LDH-Ca_2_Al nanocomplexes exhibited a reduced content of hydroxyl groups with pro-CO_2_ nature exposed on the surface of LDH-Ca_2_Al, improving the interfacial interaction between the nanofillers and the rubber matrix and enhancing the dispersion of GO/LDH-Ca_2_Al in the polymers. Moreover, CO_2_ in the soluble GO/LDH-Ca_2_Al nanocomposites was significantly reduced, while the diffusion properties demonstrated weak temperature dependence on solubility. The mechanism of the CO_2_ gas barrier of polymers filled with GO/LDH-Ca_2_Al was proposed on the basis of the Arrhenius equation.

## 1. Introduction

Climate change is one of the most serious problems facing mankind, and anthropogenic air pollutant emissions and climate change have a direct relationship in which carbon dioxide (CO_2_) is the main air pollutant [1]. In the field of oilfield development, CO_2_ is an attractive repellant for enhanced oil recovery, but some problems, such as CO_2_ leakage during CO_2_ transportation, are encountered; hence, a sealing material with excellent barrier performance needs to be developed [2].

Rubber is a highly elastic and amorphous polymer with good toughness, elasticity, and elongation, and they are also known as elastomers; for this class of material, a very small external force can produce a large deformation, which can be restored after removing the external force [3,4]. Rubber materials are widely used in tire liners, chemical protection products, medical packaging, automotive tanks and natural gas storage tanks, and natural long-distance pipeline valve sealing materials, and their gas barrier properties are very important [5]. The gas barrier properties of rubber materials are very important. Given the high free volume fraction between rubber molecular chains, gas molecules can easily diffuse from one end of the rubber material to the other, and most diene-based rubbers such as natural rubber (NR), ethylene–propylene rubber (EPDM), and styrene–butadiene rubber (SBR) have high permeability to gases [6].

The gas barrier properties can be effectively improved by filling a certain amount of nanoparticles into the rubber to form a filling network, thus creating distorted paths and inhibiting gas molecules from penetrating the rubber matrix. More importantly, the strong interface between the nanoparticles and the rubber molecules is significant in limiting chain migration and further reducing the free volume between the nanoparticles and the rubber molecules. Among a range of nanoparticles, layered particles such as layered double hydroxides (LDH) [7,8,9] and graphene (GE) or graphene oxide (GO) [10,11] have higher aspect ratios than spherical and fibrous particles, making them more conducive to enhancing the gas barrier properties of rubber composites [12]. In particular, LDH is considered to be a promising CO_2_ adsorbent because of its controllable layer spacing and pro-CO_2_ properties [13]. The chemical formula of LDH can be represented by the general expression $M_{1-x}^{2+}M_{x}^{3+}{\left(OH\right)}_{2}{\left(A^{n-}\right)}_{x/n}\cdot yH_{2}O$ [14,15]. LDH consists of a hydromagnesite-like layer in which a small portion of octahedrally coordinated divalent metal cations is replaced by trivalent metal cations, resulting in a positively charged host layer [16]. Exchangeable inorganic or organic anions are accommodated in the interlayer channels to compensate for the positive charge. In addition, the hydroxyl groups of the host layer are connected to anions or water molecules via hydrogen bonding. The *X* value is equal to the molar ratio of M^3+^/(M^2+^+M^3+^), and it ranges from 0.17 to 0.33 [16]. Given the controllable M^2+^/M^3+^ molar ratio, the tunability of the metal cations, and the exchangeable charge compensating anions [17], the layer spacing of the LDH can be optimized, which can aid in the design of the lamellar packing that facilitates CO_2_ intercalation, further forming a more complex pathway for CO_2_ diffusion.

However, the thermal stability of LDH is poor, and it starts to undergo layer structure destruction at approximately 250 °C [18]. Most importantly, LDH is a polar inorganic material with limited compatibility with organic macromolecules such as natural rubber (NR), fluorine rubber (FKM), and nitrile rubber (NBR), leading to agglomeration in the polymer and the formation of weak phase interfaces [8,9]. Therefore, the dispersion and thermal stability of LDH in polymers can be improved by forming a compound with LDH along with new components. GO has high electronic conductivity, good mechanical strength, excellent thermal stability, large specific area, and abundant oxygen-containing groups on its surface, such as epoxide, hydroxyl, and carboxyl groups, which can form strong interactions with polymer molecules via hydrogen or ionic bonding [13,14,15]. It also has good descriptive properties with polymers. Moreover, GO is an almost monolayer layered structure with a large surface area [19]. GO is an ideal carrier for LDH because of its negatively charged layer surface and oxygen-containing groups, which can form hydrogen bonding and strong electrostatic interactions with the positively charged LDH on the surface of the host layer [20,21,22]. Yang et al. prepared FeNi-LDH/GO hybrid nanosheets by alternately stacking GO layers and FeNi double hydroxide ion layers [20].

To meet the application requirements for rubber of the tire, aerospace, and military fields, nanofillers such as graphene oxide, carbon nanotubes, carbon black, and montmorillonite have been widely used to improve the mechanical properties of rubber. The effects of various modifying additives and fillers on the physicochemical and mechanical properties of polymer composites are determined by many factors as follows: (1) filler–polymer interfacial interaction [23,24,25], (2) content of fillers [26], and (3) cross-linking density of polymers [27]. Among these, filler–polymer interfacial interaction exerts an extremely significant effect on the mechanical properties of polymer composites.

**Scheme 1:**
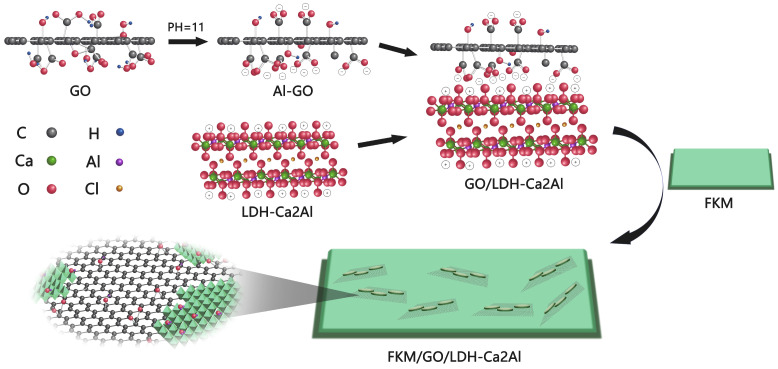
Preparation flow of GO/LDH-Ca_2_Al nanocomposite fillers.

In this study, GO/LDH-Ca_2_Al nanolamellar fillers with better thermal stability and better compatibility with polymers were prepared via a simple synthesis method (Scheme 1). The layer spacing of this nanofiller increased by approximately 0.04 nm compared with that of pristine LDH-Ca_2_Al, thus increasing the chances of interlayer insertion of CO_2_ gas molecules. At the same time, the resistance to CO_2_ stability was significantly improved, and a stable layer structure can be maintained in a CO_2_ environment. The GO/LDH-Ca_2_Al nanofiller reduced the content of exposed hydroxyl groups of a pro-CO_2_ nature on the surface of LDH-Ca_2_Al, increased the interfacial interactions between LDH-Ca_2_Al and the rubber matrix, and improved the dispersion of LDH-Ca_2_Al in the polymer. Consequently, the CO_2_ solubility of the nanocomposites was significantly reduced.

## 2. Experimental

### 2.1. Materials

FKM 246, a terpolymer of vinylidene fluoride (VF2); hexafluoropropylene (HFP); and tetrafluoroethylene (TFE), with 68.5% fluorine content, density of 1.86 g/cm^3^, Mooney viscosity, ML 1 + 10 at 121 °C = 25, and solubility parameter, δ = 19.7 MPa^1/2^ were supplied by Shanghai Huayi 3F New Materials Co., Ltd. (Shanghai, China). 2,5-Dimethyl-2,5-di(tert-butylperoxy)hexane (Macklin), triallyl isocyanurate (TAIC, ≥98%, Aladdin), and calcium chloride dihydrate (CaCl_2_·2H_2_O, ≥98%) were purchased from Beijing Yongchang Haoran Bio-technology Co., Ltd. (Beijing, China). Aluminum chloride hexahydrate (AlCl_3_·6H_2_O, ≥98%) was obtained from Shanghai Taitan Technology Co., Ltd. (Shanghai, China). Graphene oxide (GO, >99%), sodium hydroxide (NaOH, ≥98%), and deionized water were purchased from Shanghai Boer Chemical Reagent Co. (Shanghai, China). All reagents were of analytical grade, and the purity of the CO_2_ test gas used for the experiments was 99.9% (Beijing Chengweixin Industrial Gas Sales Center, Beijing, China).

### 2.2. Preparation of Layered LDH-Ca_2_Al and GO/LDH-Ca_2_Al

LDH-Ca_2_Al was prepared via co-precipitation [28,29,30]. Approximately 15 g CaCl_2_·2H_2_O and 7.606 g AlCl_3_·6H_2_O were dissolved in 300 mL of deionized water to obtain a colorless and transparent Ca_2_Al solution. Approximately 7.36 g NaOH was dissolved in 30 mL of deionized water to obtain a colorless and transparent NaOH solution, and the NaOH solution was added to the vigorously stirred (800 rpm) Ca_2_Al solution at room temperature. The pH of the suspension was maintained at approximately 11, and stirring was maintained for 1 h. The sample was then filtered, washed with deionized water until the pH was approximately 10, and finally vacuum-dried at 60 °C to obtain the white powder LDH-Ca_2_Al.

Approximately 0.5 g GO was added to 1050 mL of deionized water to obtain the GO suspension [31]. Then, an appropriate amount of NaOH was added to adjust the pH of GO suspension at approximately 11 (Al-GO), and 8.15 g LDH-Ca_2_Al was added to the solution. The sample was sonicated for 10 min and then placed in a vacuum oven at 60 °C for 24 h to obtain GO/LDH-Ca_2_Al suspension. Finally, it was filtered, washed with deionized water, and freeze-dried.

### 2.3. CO_2_ Resistance Stability Testing of LDH-Ca_2_Al and GO/LDH-Ca_2_Al

S-LDH-Ca_2_Al and S-GO/LDH-Ca_2_Al, which were intercalated and reacted by CO_2_, were obtained by simultaneously placing 1.5 g of LDH-Ca_2_Al and GO/LDH-Ca_2_Al in an autoclave of CO_2_ gas as the ambient medium at 80 °C and 4.3 MPa, and the sample was taken out after 1 day.

### 2.4. Preparation of FKM/GO/LDH-Ca_2_Al and FKM/LDH-Ca_2_Al Composites

The raw rubber was sheared on the rolls for 2 min at room temperature by using an open double-roller mill (friction ratio: 1:1.4). Then, different proportions of GO/LDH-Ca_2_Al were added for 3–4 min. Finally, 5 phr LUPEROX 101XL-50 and 5 phr TAIC were added to the sample, and the mixture was sheared and agitated for 3–4 min to obtain the unvulcanized FKM/GO/Ca_2_Al composite. A rotorless vulcanometer was used to determine the optimal vulcanization time (tc90) of the unvulcanized FKM/GO/LDH-Ca_2_Al composites at 160 °C, and was vulcanized to the optimal vulcanization time (tc90) by using a plate vulcanizer at 160 °C and 10 MPa. FKMs unfilled and filled with the same amount of LDH-Ca_2_Al were prepared using the same method as the blank control group. Table 1 provides the formulation of FKM/GO/LDH-Ca_2_Al composites. Different portions of GO/LDH-Ca_2_Al-filled fluoroelastomers were denoted as FKM/GO/LDH-Ca_2_Al-*x*, where *x* refers to the number of portions of nanofillers in 100 phr of fluoroelastomers.

### 2.5. Characterization

Fourier transform infrared spectroscopy (FT-IR) measurements were carried out on a TENSOR II spectrometer (Bruker, MA, USA) at the wave number range of 4000–400 cm^−1^ with a resolution of 4 cm^−1^ and a 64-scan signal via the KBr pellet technique. An X-ray diffractometer (D8 Focus, Bruker) was used to characterize LDH-Ca_2_Al and GO/LDH-Ca_2_Al before and after CO_2_ immersion and GO, and the measurements were carried out at a wavelength of *λ* = 0.154056 nm with a step size of 0.01° under Cu/Kα radiation. LDH-Ca_2_Al and GO/LDH-Ca_2_Al before and after CO_2_ immersion, FKM/LDH-Ca_2_Al, and FKM/GO/LDH-Ca_2_Al were subjected to morphological observation by using a SU8010 cold field emission scanning electron microscope (SU8010, Hitachi, Japan). The CO_2_ gas permeability of FKM/LDH-Ca_2_Al and FKM/GO/LDH-Ca_2_Al composites were measured via differential pressure by using a homemade gas permeability tester according to ISO 2782-1:2022 [32]. The samples to be tested were cut into dumbbell-shaped specimens. The tensile test was carried out by using a universal testing machine at room temperature with a tensile rate of 500 mm/min, following the GB/T 528–2009 standard [33] and three parallel specimens were used for each group of materials. According to the ISO 7619-1:2010 standard [34], the Shore A hardness of the composites was tested using an LX A-type hardness tester. The samples were subjected to DMA testing by using a DMA (NETZSCH 242 C) in tensile mode at the frequency of 10 Hz. Each sample was scanned in the range of −50 °C to 60 °C at a heating rate of 3 °C/min^−1^. The samples were subjected to thermogravimetric analysis (TGA) by using a Shimadzu DTG-60 thermogravimetric analyzer, and the weight of the test samples ranged from 8 mg to 10 mg. The samples were heated from 25 °C to 800 °C at a heating rate of 10 °C/min under a nitrogen atmosphere purge of 100 mL/min. A differential scanning calorimeter (DSC, NETZSCH-5) was used to characterize the LDH-Ca_2_Al and GO/LDH-Ca_2_Al thermal transition. The weight of the test samples ranged from 6 mg to 10 mg and was heated from 25 °C to 250 °C at a heating rate of 10 °C/min under a nitrogen atmosphere of 100 mL/min.

The crosslink density of FKM/LDH-Ca_2_Al and FKM/GO/LDH-Ca_2_Al was measured using the equilibrium dissolution method [27,35] according to the standard ASTM D6814-02 [36]. Approximately 0.2 g of the sample was placed in a sealed container containing 25 mL of acetone and held at 25 °C for 3 days, during which the acetone solvent was changed every 24 h. The mass of the swelling equilibrium was measured as $m_{S}$ and dried in a blower oven at 70 °C for 16 h. Then, the weight of the dried rubber $m_{d}$ was measured, and the density $\rho_{d}$ was calculated. The crosslink density $v_{e}$ was calculated as follows:

(1) $$V_{r}=(m_{d}/\rho_{d})/\left[m_{d}/\rho_{d}+\left(m_{S}-m_{d}\right)/\rho_{S}\right]$$

(2) $$v_{e}=\frac{-\left[ln\left(1-V_{r}\right)+V_{r}+\chi_{1}{V_{r}}^{2}\right]}{\left[V_{1}({V_{r}}^{1/3}-V_{r})/2\right]}$$

where $V_{r}$ and $\rho_{S}$ are the volume fraction of rubber and the density of the solvent (the density of acetone is 0.79 g/cm^3^), respectively, $V_{1}$ is the molar volume of acetone solvent (73.53 cm^3^/mol), and $\chi_{1}$ is the interaction parameter of the polymer with the solvent, which is 0.358 in this case. According to the Bristow–Watson equation, the following expression can be obtained [37]:

(3) $$\chi_{1}=\beta_{1}+\left(\frac{V_{1}}{RT}\right){\left(\delta_{s}-\delta_{p}\right)}^{2}$$

where $\beta_{1}$ is the lattice constant (typically 0.34), R is the gas constant, T is the absolute temperature, and $\delta_{p}$ and $\delta_{s}$ are the solubility parameters of FKM and solvent acetone, respectively.

## 3. Results and Discussion

### 3.1. Fourier Transform Infrared (FTIR) Spectroscopy

**Figure 1:**
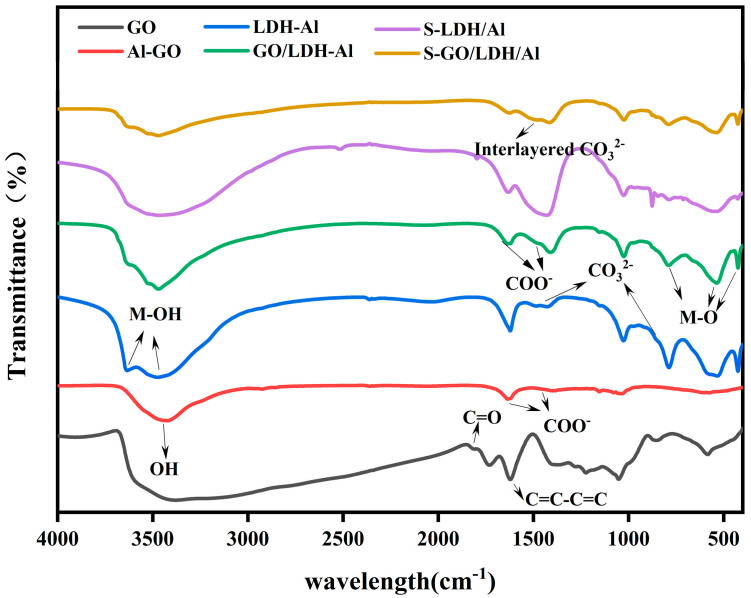
IR spectra of GO, Al-GO, LDH-Ca_2_Al, GO/LDH-Ca_2_Al, S-LDH-Ca_2_Al, and S-GO/LDH-Ca_2_Al.

The IR spectra of GO, Al-GO, LDH-Ca_2_Al, GO/LDH-Ca_2_Al, S-LDH-Ca_2_Al, and S-GO/LDH-Ca_2_Al are shown in Figure 1. The broad and strong absorption peaks of the infrared spectra of GO near 3381 cm^−1^ represent the O–H stretching vibrations in carboxyl and hydroxyl groups, and the peaks at 1223 and 1733 cm^−1^ represent the out-of-plane bending vibrations of O–H in COOH and its C=O stretching vibrations [38,39]. Alkoxy (C–OH) stretching vibrations and hydroxyl deformation vibrations occurred at 1052 and 1390 cm^−1^, respectively [40], and while that at 1620 cm^−1^ can be attributed to the conjugate bond of aromatic C=C-C=C [39]. The peaks of the C=O stretching vibration of COOH and O–H out-of-plane bending vibration of COOH on Al-GO disappeared, and the symmetric and antisymmetric peaks of COO^−^ appeared near 1636 and 1458 cm^−1^ [41]. The O–H stretching vibration peaks of Al–OH and Ca–OH of LDH-Ca_2_Al, GO/LDH-Ca_2_Al, S-LDH-Ca_2_Al, and S-GO/LDH-Ca_2_Al appeared at 3634 and 3474 cm^−1^, respectively, the bending vibration of the interlayer H_2_O molecule was observed at approximately 1621 cm^−1^, and the absorption peaks at 787, 535, and 434 cm^−1^, which are below 800 cm^−1^, represent M-O vibrational peaks (M is Ca or Al) [18,41,42]. In the IR spectra of GO/LDH-Ca_2_Al, Al-GO peaks appeared at 3741 and 1637 cm^−1^, but these peaks appear to be blue-shifted, possibly because of the positively charged nature on the LDH-Ca_2_Al layer and the presence of the electron-absorbing induced effect. The peaks at 1733 and 1223 cm^−1^, which represent COOH, disappeared from the infrared spectra of GO/LDH-Ca_2_Al and Al-GO, while symmetric and antisymmetric telescopic vibrational peaks near 1458 and 1636 cm^−1^ for COO^−^ appeared [41]. The symmetric and antisymmetric telescopic vibrational peaks of COO^−^ in the GO/LDH-Ca_2_Al spectra also shifted to the high-frequency region, indicating the presence of a strong interaction between GO and LDH-Ca_2_Al.

The CO_3_^2−^ peaks at 1428 and 875 cm^−1^ represent small amounts of CaCO_3_ impurities in GO/LDH-Ca_2_Al with LDH-Ca_2_Al [43]. Both LDH-Ca_2_Al and GO/LDH-Ca_2_Al showed weak interlayer CO_3_^2−^ absorption peaks at 2974 and 1486 cm^−1^, but the interlayer CO_3_^2−^ absorption peaks of LDH-Ca_2_Al of S-LDH-Ca_2_Al and S-GO/LDH-Ca_2_Al were significantly enhanced, suggesting that CO_2_ can be intercalated in the interlayer of LDH-Ca_2_Al mainly in the form of CO_3_^2−^ [18].

### 3.2. XRD Analysis of LDH-Ca_2_Al and GO/LDH-Ca_2_Al

**Figure 2:**
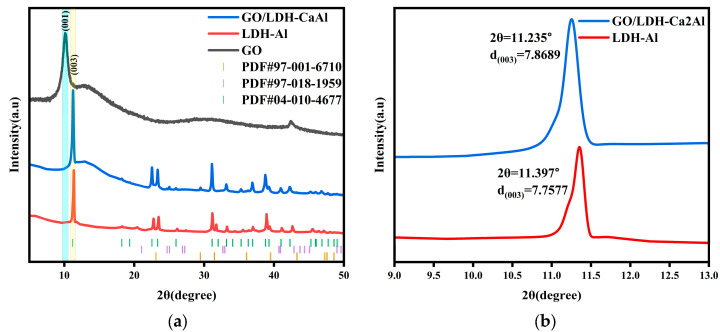
(**a**) XRD of GO, LDH-Ca_2_Al, and GO/LDH-Ca_2_Al and (**b**) localized enlargement near (003) of LDH-Ca_2_Al and GO/LDH-Ca_2_Al.

The XRD results of GO, LDH-Ca_2_Al, and GO/LDH-Ca_2_Al are shown in Figure 2, where the appearance of CaCO_3_ impurity (PDF #97-001-6710 and PDF #97-018-1959) peaks proves the accuracy of the FITR results (Figure 1). The X-ray diffraction pattern of GO/LDH-Ca_2_Al (PDF#04-010-4677) does not show GO diffraction peaks, suggesting that the periodic stacking arrangement was not formed by the composite of GO and LDH-Ca_2_Al [13]. The GO/LDH-Ca_2_Al shows the typical Bragg reflection of LDH-Ca_2_Al as the pristine LDH-Ca_2_Al, but the diffraction peaks of GO/LDH-Ca_2_Al at the (003) crystal plane shifted to a lower angle; according to Bragg equation ($2dsin\left(2\theta \right)=n\lambda$) the calculated LDH-Ca_2_Al and GO/LDH-Ca_2_Al basal spacings were approximately 2.587 and 2.623 nm, respectively, and the thickness of the LDH-like hydromagnesite layer was 0.48 nm [41]. The LDH-Ca_2_Al and GO/LDH-Ca_2_Al interlayer spacings were 2.107 and 2.143 nm, respectively. Consequently, Al-GO formed a complex with LDH-Ca_2_Al, resulting in an increase in interlayer spacing CO_2_ by approximately 0.036 nm, as shown in Figure 2b. This phenomenon can be attributed to the strong interactions between the Al-GO and the LDH-Ca_2_Al layers, which weakened the electrostatic interactions of the anionic in the interlayer channel with the cationic on the layer, consistent with the FTIR results. Given the increase in GO/LDH-Ca_2_Al layer spacing, the specific surface area also increased, which provided more active sites for CO_2_ molecular adsorption and increased the possibility of CO_2_ gas adsorption and intercalation.

### 3.3. Structural Characteristics of LDH-Ca_2_Al and GO/LDH-Ca_2_Al

**Figure 3:**
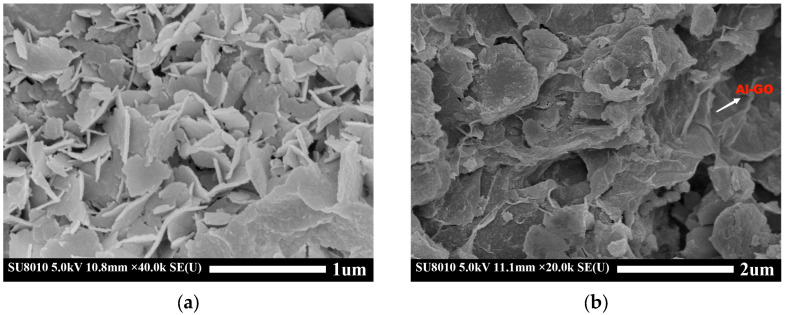
SEM images of (**a**) LDH-Ca_2_Al and (**b**) GO/LDH-Ca_2_Al.

**Figure 4:**
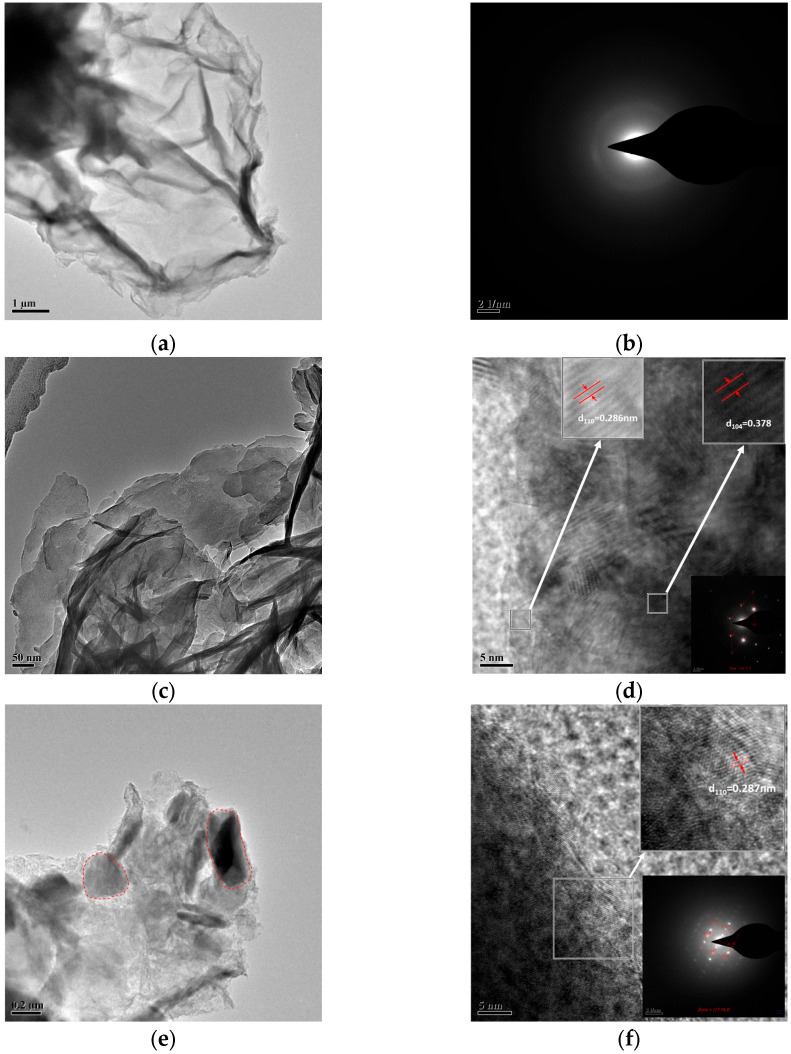
TEM of (**a**) GO, (**c**) LDH-Ca_2_Al, and (**e**) GO/LDH-Ca_2_Al (red circles—LDH-Ca_2_Al), (**b**) SAED diagrams of GO, and (**d**) lattice fringes of LDH-Ca_2_Al and (**f**) GO/LDH-Ca_2_Al with SAED plots inset in the lower left corner.

The SEM of images of LDH-Ca_2_Al and GO/LDH-Ca_2_Al are shown in Figure 3. LDH-Ca_2_Al and GO/LDH-Ca_2_Al have obvious lamellar structures, and the diameter of LDH-Ca_2_Al flakes is in the range of 0.3–0.5 µm with a thickness of approximately 0.03 µm. The XRD of LDH-Ca_2_Al showed a layer spacing of approximately 2.107 nm (Figure 2), indicating that LDH-Ca_2_Al is composed of multilayered LDH-Ca_2_Al lamellae stacked along the (003) direction. In GO/LDH-Ca_2_Al, LDH-Ca_2_Al lamellae are much smaller than Al-GO (S1), which is consistent with the TEM results of GO/LDH-Ca_2_Al in Figure 4e. A closer look reveals that a large amount of LDH-Ca_2_Al is loaded onto the surface of Al-GO because of the existence of a strong interaction force between LDH-Ca_2_Al and Al-GO, which is consistent with the FITR results of GO/LDH-Ca_2_Al (Figure 1).

The TEM of GO, LDH-Ca_2_Al, and GO/LDH-Ca_2_Al is shown in Figure S1 and Figure 4, and they show a lamellar structure, which is consistent with the SEM results. The HR-TEM of LDH-Ca_2_Al in Figure 4d shows the appearance of obvious (110) and (104) lattice stripes, in which the spacing of the stripes are 0.286 and 0.378 nm, consistent with the results of (110) and (104) diffraction peaks in the XRD diffraction pattern. The SAED plot along the crystal axis (16, −7, 1) is shown in the lower right corner of Figure 4d, consistent with PDF#04-010-4677. A large amount of two-dimensional LDH-Ca_2_Al grows on GO/LDH-Ca_2_Al, and the HR-TEM of GO/LDH-Ca_2_Al clearly demonstrates that the two-dimensional GO in GO/LDH-Ca_2_Al grows LDH-Ca_2_Al (Figure 4e); a (110) lattice face of LDH-Ca_2_Al was observed in the GO/LDH-Ca_2_Al lattice streak, and the SAED image in this region shows LDH-Ca_2_Al diffraction spots along the (17,16,2) directions [44], indicating that LDH-Ca_2_Al is deposited on the GO surface and stacked along the (003) direction on the GO surface.

### 3.4. Thermal Stability of GO, LDH-Ca_2_Al, and GO/LDH-Ca_2_Al

**Figure 5:**
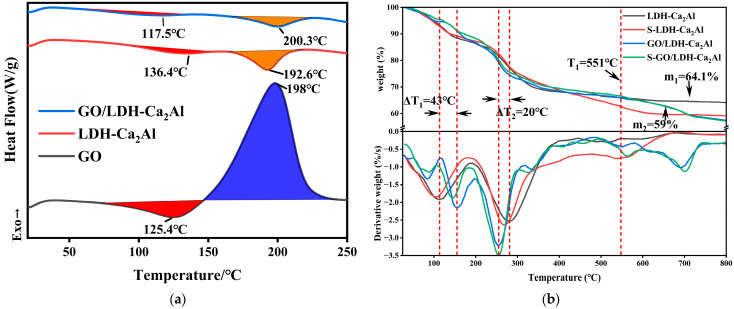
(**a**) DSC curves of GO, LDH-Ca_2_Al, and GO/LDH-Ca_2_Al and (**b**) TGA-DTG curves of LDH-Ca_2_Al, S-LDH-Ca_2_Al, GO/LDH-Ca_2_Al, and S-GO/LDH-Ca_2_Al.

The DSC and TGA curves of GO, LDH-Ca_2_Al, and GO/LDH-Ca_2_Al are shown in Figure 5a–c, respectively. Figure 5a shows a physisorbed water removal near 125.4, 136.4, and 117.5 °C for GO, LDH-Ca_2_Al, and GO/LDH-Ca_2_Al, respectively. Meanwhile, GO shows the oxidation of oxygen-containing functional groups near 198 °C, indicating an exothermic reaction [45]. The removal of interlayer H_2_O molecules from LDH-Ca_2_Al and GO/LDH-Ca_2_Al occurred near 192.6 and 200.3 °C, respectively, suggesting that GO/LDH-Ca_2_Al has better thermal stability than LDH-Ca_2_Al. Meanwhile, GO/LDH-Ca_2_Al did not show the exothermic reaction of GO near 198 °C, indicating that the thermal stability of GO in GO/LDH-Ca_2_Al was significantly improved due to the presence of strong interaction between Al-GO and LDH-Ca_2_Al.

**Figure 6:**
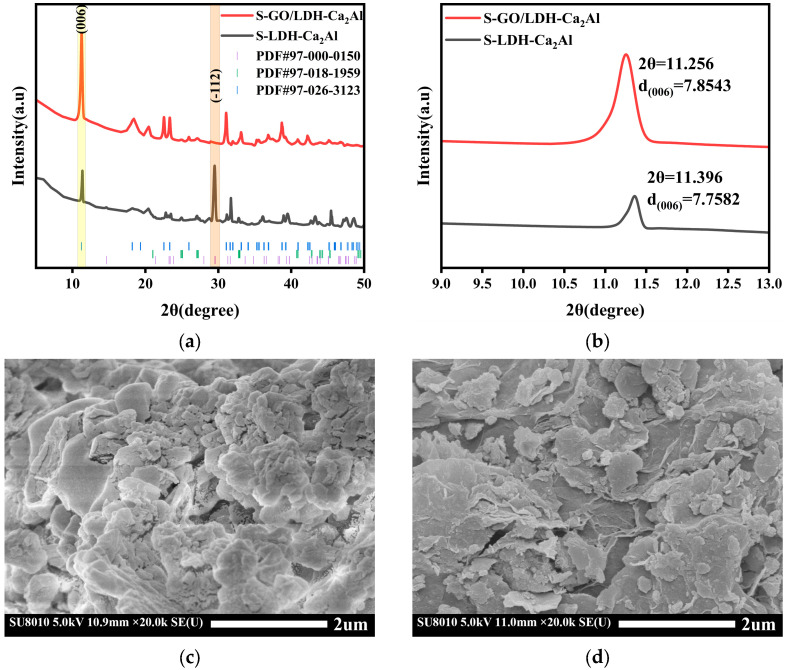
(**a**) XRD of S-LDH-Ca_2_Al, S-GO/LDH-Ca_2_Al, and (**b**) localized enlargement near (003) of LDH-Ca_2_Al and GO/LDH-Ca_2_Al. (**c**) SEM of S-LDH-Ca_2_Al, and (**d**) S-GO/LDH-Ca_2_Al.

The thermal stability of GO, LDH-Ca_2_Al, and GO/LDH-Ca_2_Al is also depicted in Figure S2 and Figure 5b. Similar to the DSC results (Figure 5a), GO underwent three key weight loss steps at <125 °C corresponding to the removal of physically adsorbed water, 130–350 °C corresponding to the removal of oxygen functional groups, and 360–800 °C corresponding to the carbon framework of the oxidative pyrolysis [39]. Based on Figure 5b, three distinct weight loss steps can be observed for LDH-Ca_2_Al at 30–100 °C corresponding to the loss of physisorbed water, 100–170 °C corresponding to the process of interlayer water removal, 215–400 °C corresponding to the dehydroxylation of the layers and the removal of interlayer chloride ions, and 440–550 °C corresponding to the further elimination of the hydroxyl groups on the layers [18,41,46]. Compared with LDH-Ca_2_Al, the thermal stability of GO/LDH-Ca_2_Al was significantly improved between 30 and 200 °C. Although the temperature at which approximately 1.2% of physisorbed water was lost was approximately 7 °C lower than that of LDH-Ca_2_Al, the temperature at which the interlayer water was removed increased by nearly 43 °C (Δ*T*_1_). Interestingly, the temperature of releasing interlayer chloride ions decreased by about 20 °C (Δ*T*_2_), which was caused by the enlarged interlayer spacing of LDH-Ca_2_Al. At the same time, the thermal stability of the GO layer on GO/LDH-Ca_2_Al was improved, in which the carbon framework decomposition temperature increased by approximately 29 °C, respectively. The residual mass of S-LDH-Ca_2_Al with a value of approximately 59% at 800 °C (*m*_2_), which is approximately 5% higher than that of the LDH-Ca_2_Al, can be attributed to the decomposition of the carbonates that occurred after 450 °C based on the XRD patterns of S-LDH-Ca_2_Al; this finding revealed the presence of carbonates (Figure 6a) [18].

### 3.5. Carbon Dioxide Resistance Stability of LDH-Ca_2_Al and GO/LDH-Ca_2_Al

The XRD of S-LDH-Ca_2_Al and S-GO/LDH-Ca_2_Al are shown in Figure 6a,b. LDH-Ca_2_Al and GO/LDH-Ca_2_Al were placed in a pure CO_2_ environment at a pressure of 4 MPa and a temperature of 80 °C. After 1 day, the XRD diffraction peaks of the reaction products S-LDH-Ca_2_Al and S-GO/LDH-Ca_2_Al were very similar to those of Ca_4_Al_2_ (OH)_12_ (OH_0_._4_ (CO_3_)_0.8_ (H_2_O)_4_ compounds (PDF#97-026-3123). According to the Bragg equation ($2dsin\left(2\theta \right)=n\lambda$), the calculated layer spacings of LDH-Ca_2_Al, GO/LDH-Ca_2_Al, S-LDH-Ca_2_Al, and S-GO/LDH-Ca_2_Al were 2.106, 2.143, 2.106, and 2.138 nm, respectively (Figure 2b and Figure 6b) [41], and the kinetic diameter of CO_2_ was approximately 0.33 nm [47]. The IR spectra of S-LDH-Ca_2_Al and S-GO/LDH-Ca_2_Al also showed significant interlayer CO_3_^2−^ absorption peaks (PDF #97-018-1959 and PDF #97-000-0150, Figure 1), suggesting that CO_2_ was intercalated into the interlayer of LDH and appeared in the interlayer in the form of CO_3_^2−^. The intensity of the CaCO_3_ diffraction peaks increased in the S-LDH-Ca_2_Al, and the micro-morphology of the S-LDH-Ca_2_Al showed a large number of massive structures (Figure 6c), indicating that CO_2_ reacted with LDH-Ca_2_Al, which led to an increase in CO_3_^2−^ content in S-LDH-Ca_2_Al. By contrast, the intensity of the CaCO_3_ diffraction peaks in the XRD pattern of S-GO/LDH-Ca_2_Al decreased, no significant change was observed in the microscopic morphology (Figure 6d), and GO/LDH-Ca_2_Al hardly reacted with CO_2_, indicating that GO/LDH-Ca_2_Al has a very good CO_2_-resistant chemical stability, which is consistent with the TGA results (Figure 5). Table 2 also provides information on the change of CaCO_3_ content in LDH-Ca_2_Al, GO/LDH-Ca_2_Al, S-LDH-Ca_2_Al, and S-GO/LDH-Ca_2_Al.

### 3.6. Interfacial Interactions of FKM Matrix with LDH-Ca_2_Al and GO/LDH-Ca_2_Al

**Figure 7:**
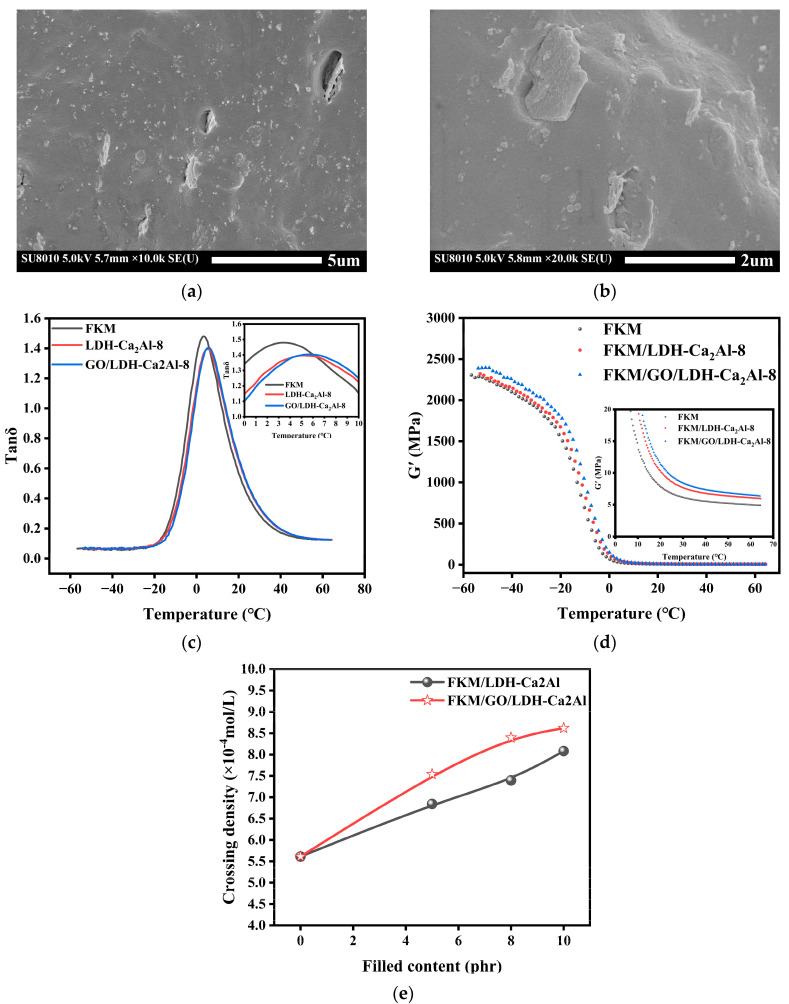
SEM images of (**a**) FKM/LDH-Ca_2_Al-8 and (**b**) FKM/GO/LDH-Ca_2_Al-8, (**c**,**d**) DMA of FKM, LDH-Ca_2_Al-8, and GO/LDH-Ca_2_Al-8, and (**e**) Crosslink density of FKM/LDH-Ca_2_Al and FKM/GO/LDH-Ca_2_Al with different filler parts.

**Figure 8:**
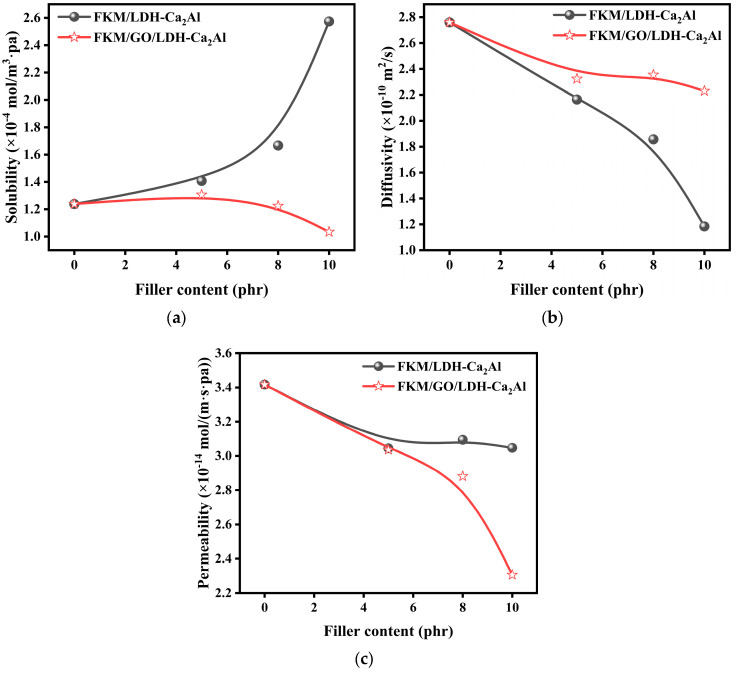
(**a**) Solubility (S), (**b**) diffusion coefficient (D), and (**c**) permeability coefficient (Q) of FKM, FKM/GO/LDH-Ca_2_Al, and FKM/LDH-Ca_2_Al as a function of filler content.

The interaction between the nanofillers and the polymer matrix influences the dispersion state of the nanofillers in the polymer, thus remarkably affecting the gas barrier properties and mechanical properties of the nanocomplexes [48,49,50]. GO/LDH-Ca_2_Al nanofillers had an excellent dispersion state in the FKM/GO/LDH-Ca_2_Al nanocomposites, and the energy storage modulus of the FKM/GO/LDH-Ca_2_Al complexes substantially increased because of the existence of a good phase interface between the GO/LDH-Ca_2_Al nanofillers and the rubber matrix compared with the LDH-Ca_2_Al nanofillers (Figure 7a,b). This interfacial interaction will be quantitatively measured next using two-phase modeling [51]. The loss tangent relationship between the filler and the unfilled polymer matrix satisfies the following equation [27,51,52]:

(4) $$tan\;\delta =\frac{tan\;\delta_{m}}{1+1.5B\varphi }$$

where $tan\;\delta_{m}$ and tan δ represent the tangent values of the filler and the unfilled polymer matrix, φ is the volume fraction of the filler, and B is a phenomenological interaction parameter, which can represent the strength of the interface interaction between the filler and the matrix; the larger the value of B, the stronger the interface interaction between the filler and the polymer matrix [27]. The $tan\;\delta_{m}$, tan δ, *T_g_*, and B values of FKM, FKM/LDH-Ca_2_Al-8, and FKM/GO/LDH-Ca_2_Al-8 are shown in Table 3. The loss tangent tan δ and *T_g_* of FKM/GO/LDH-Ca_2_Al shifted toward high temperature, and the B value of FKM/GO/LDH-Ca_2_Al-8 is approximately 0.528, which is slightly higher than that of FKM/LDH-Ca_2_Al-8 by 0.041. This finding indicates that the interfacial interactions between GO/LDH-Ca_2_Al and the FKM matrix were enhanced compared with that of LDH-Ca_2_Al, thus hindering the movement of the molecular chain segments. this finding explains that the crosslink density of FKM/GO/LDH-Ca_2_Al is always higher than that of FKM/LDH-Ca_2_Al for the fluoroelastomer composites filled with the same content of fillers, as shown in Figure 8. The SEM images of FKM/LDH-Ca_2_Al-8 and FKM/GO/LDH-Ca_2_Al-8 are shown in Figure 7a,b. The results indicate a good phase interface between GO/LDH-Ca_2_Al and the rubber matrix, which is caused by the strong interfacial interaction between GO/LDH-Ca_2_Al and the fluoroelastomer matrix than that of LDH-Ca_2_Al.

### 3.7. Gas Barrier Properties of FKM/LDH-Ca_2_Al and FKM/GO/LDH-Ca_2_Al Composites

#### 3.7.1. Effect of Filler Content on the Gas Barrier Properties of FKM/LDH-Ca_2_Al and FKM/GO/LDH-Ca_2_Al Composites

The gas barrier properties of rubber composites, which were measured in terms of the gas permeability coefficient (Q=DS), are related to the shape, content, and degree of dispersion of the filler [5,12,53], and lamellar fillers with large aspect ratios have good gas barrier properties [54]. LDH-Ca_2_Al and GO/LDH-Ca_2_Al are lamellar fillers with large aspect ratios (Figure 3a,b). The aspect ratios of LDH-Ca_2_Al are in the range of 10–16, and the Q and D of the rubber filled with 8 phr LDH-Ca_2_Al fillers were reduced by nearly 9% and 33%, respectively. The relationships of permeability coefficient (Q), diffusion coefficient (D), and solubility (S) with the contents of LDH-Ca_2_Al and GO/LDH-Ca_2_Al are illustrated in Figure 8a–c. The results show that the permeability coefficients and diffusion coefficients of the rubber composites decrease significantly with the increase in filler content, which is consistent with the change in the crosslinking density of the rubber composites (Figure 7e). This phenomenon can be explained using the free volume theory [55,56]. The nanofillers can be approximated as physical cross-linking points and the cross-linking density increases with the increase in nanofiller content. The increase in cross-linking density shortens the distance between the rubber chains, reduces the mobility of the rubber chains, and leads to a decrease in the free volume fraction of the permeation path of the gas molecules [6]. The free volume fraction of the gas molecules’ permeation path decreased.

The variation of solubility (*S*) with filler content is given in Figure 8a. The solubility of FKM/LDH-Ca_2_Al increased significantly with the increase in LDH-Ca_2_Al content, and the solubility of the rubber composite increased by 108% when filled with 10 phr of LDH-Ca_2_Al. By contrast, the solubility of FKM/GO/LDH-Ca_2_Al gradually decreased with the increase in GO/LDH-Ca_2_Al content, and the solubility of the rubber composite decreased by about 19% when filled with 10 phr of GO/LDH-Ca_2_Al. This finding was obtained because LDH-Ca_2_Al is a nanofiller with high CO_2_ absorption [48]. Moreover, the loading of LDH-Ca_2_Al onto the GO surface reduces the content of exposed polar hydroxyl groups on the LDH-Ca_2_Al layer, thus forming strong hydrogen bonding interactions with O in the CO_2_ molecule and increasing the content of CO_2_ adsorbed on the surface of the filler. In addition, compared with the LDH-Ca_2_Al, the GO/LDH-Ca_2_Al in the composites has good compatibility (Figure 7a,b), which consistently supports the interfacial interactions between GO/LDH-Ca_2_Al and the rubber matrix (Table 3). Therefore, more constrained polymer regions are present near the interface between the rubber matrix and the filler [5,57], thus reducing the adsorption capacity of CO_2_ molecules at the interface.

Figure 8b shows the schematic relationship between filler content and diffusion coefficient, in which the diffusion coefficient of the rubber composites decreased significantly with the increase in the filler content of LDH-Ca_2_Al and GO/LDH-Ca_2_Al, which is consistent with many previous findings [5,58,59,60,61]. This finding was obtained because the filler increases the gas molecule diffusion path and hinders the gas molecule movement [6], while LDH-Ca_2_Al and GO/LDH-Ca_2_Al are multilayer fillers (Figure 2, Figure 3 and Figure 4) with a layer spacing of more than 2.1 nm, which is significantly higher than the kinetic diameter of the CO_2_ molecule (~0.33 nm). Hence, the CO_2_ molecule can be interpolated into the layer spacing of the filler, thus significantly improving the gas molecule diffusion path. Accordingly, the diffusion coefficient of the rubber composite material was significantly reduced. Moreover, when the filler content of the filler reached 10 phr, the diffusion coefficient of the rubber composite material was reduced by nearly 57%. However, the diffusion coefficient of FKM/LDH-Ca_2_Al is lower than that of FKM/GO/LDH-Ca_2_Al at the same filler content, in which even the diffusion coefficient of FKM/LDH-Ca_2_Al-10 is approximately 47% lower than that of FKM/GO/LDH-Ca_2_Al-10. This finding was obtained because CO_2_ entered the spacing of the LDH-Ca_2_Al layers and reacted with the LDH-Ca_2_Al to generate inorganic compounds containing CO_3_^2−^ (Table 2), such as CaCO_3_. Consequently, the diffusion activation energy of CO_2_ gas remarkably decreased during diffusion in FKM/LDH-Ca_2_Al (Table 4). For example, the diffusion activation energy of FKM/LDH-Ca_2_Al-8 increased by approximately 27.6 J/mol. However, GO/LDH-Ca_2_Al has better-stabilizing properties during CO_2_ diffusion (Figure 2, Figure 3b and Figure 6), and the CaCO_3_ content of S-GO/LDH-Ca_2_Al did not change significantly compared with that of GO/LDH-Ca_2_Al (Table 2). Hence, although FKM/GO/LDH-Ca_2_Al lost some of the ability to reduce the diffusion coefficient brought by LDH-Ca_2_Al, it improved the CO_2_ resistance stability of FKM/GO/LDH-Ca_2_Al.

#### 3.7.2. Effect of Temperature on the Gas Barrier Properties of FKM/LDH-Ca_2_Al and FKM/GO/LDH-Ca_2_Al Composites

**Figure 9:**
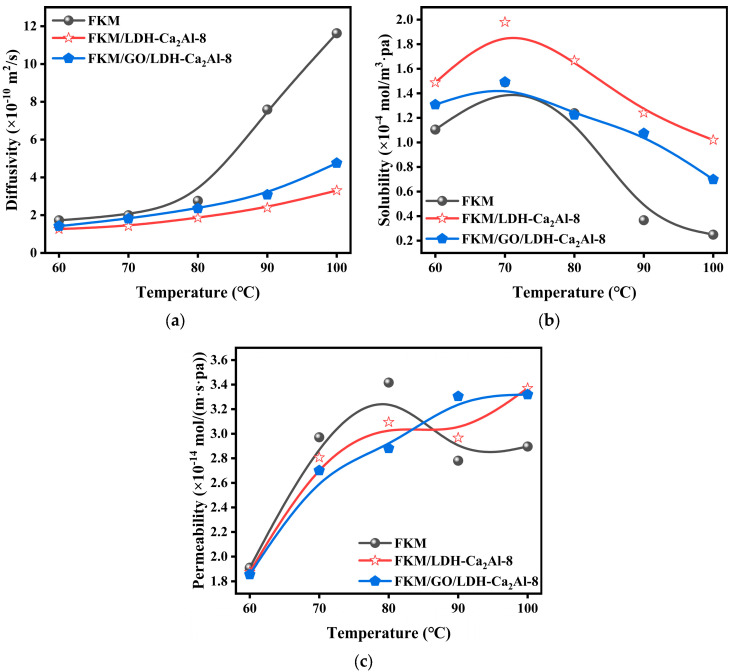
(**a**) Diffusion coefficients (D), (**b**) solubility (S), and (**c**) permeability coefficients (Q) as a function of temperature for FKM, FKM/GO/LDH-Ca_2_Al-8, and FKM/LDH-Ca_2_Al-8.

The barrier mechanism of GO/LDH-Ca_2_Al and LDH-Ca_2_Al nanofillers in FKM/GO/LDH-Ca_2_Al with FKM/LDH-Ca_2_Al composites against CO_2_ was investigated by conducting a temperature-dependence study on the gas barrier properties of FKM/LDH-Ca_2_Al and FKM/GO/LDH-Ca_2_Al composites. The permeability coefficients (Q), diffusion coefficients (D), and solubility (S) of the rubber nanocomposites with temperature are given in Figure 9. The diffusion coefficients of FKM, FKM/GO/LDH-Ca_2_Al-8, and FKM/LDH-Ca_2_Al-8 remarkably increased with temperature, but the solubility remarkably decreased with the increase in temperature. Based on this phenomenon, the Arrhenius equation can be used to explain the relationship between the ln(*S*) and ln(*D*) of rubber nanocomposites and temperature (*T*), which can be expressed by Equations (5) and (6) as follows:

(5) $$Ln\left(S\right)=Ln\left(S_{0}\right)+(∆H_{S0}+∆H_{sf}+∆H_{sc})/RT$$

(6) $$Ln\left(D\right)=Ln\left(D_{0}\right)+(∆E_{D0}+∆E_{Df}+∆E_{Dc})/RT$$

where $S_{0}$ and $D_{0}$ are pre-finger factors, which are related to the nature of the nanocomposites; $∆H_{S0}$ and $∆E_{D0}$ represent the heat of dissolution and diffusion activation energy of the pure polymer; $∆H_{sf}$ and $∆E_{Df}$ is the packing effect caused by nanofillers, which are related to the interaction of nanofillers with polymers and diffusion gases; and $∆E_{Df}$ is related to factors such as the aspect ratio, size, and shape of the nanofiller. In this experiment, $∆H_{sc}$ refers to the change in the heat of dissolution caused by the compound effect of GO and LDH-Ca_2_Al in GO/LDH-Ca_2_Al. $∆E_{Dc}$ is the activation energy of the reaction between the nanofiller and the diffusion gas. Table 4 summarizes the relevant parameters of Equations (5) and (6) for the FKM, FKM/GO/LDH-Ca_2_Al-8, and FKM/LDH-Ca_2_Al-8 samples. The diffusion activation energy (Δ*E*) of the polymers is less than −25 KJ/mol, and the solubility activation energy (Δ*H*) is higher than 12.3 KJ/mol, which is consistent with the diffusion coefficient (D) and the solubility (S) with temperature. The $∆H_{sf}$ from the packing effect of LDH-Ca_2_Al is −32.2 KJ/mol, resulting in a significant decrease in the heat of dissolution of CO_2_ in the FKM/LDH-Ca_2_Al nanocomposites to approximately 12.3 KJ/mol, which explains the strong interaction between CO_2_ and LDH-Ca_2_Al. This phenomenon led to an increase in the solubility of CO_2_ in the FKM/LDH-Ca_2_Al nanocomposites. However, the change in the heat of dissolution caused by the compound effect of GO with LDH-Ca_2_Al ($∆H_{sc}$) is 3.8 KJ/mol, which explains the existence of a strong interaction of GO/LDH-Ca_2_Al with the polymer matrix that reduced the CO_2_ adsorption effect on the nanofiller surface. The diffusion activation energy of the nanocomposites remarkably increased, which rendered the activation energy of the reaction of CO_2_ with GO/LDH-Ca_2_Al negligible, in which the value increased by approximately 42.5%. Hence, the diffusion coefficients of the FKM/LDH-Ca_2_Al and FKM/GO/LDH-Ca_2_Al nanocomposites were insensitive to the temperature variation. In addition to the packing effect caused by the filler ($∆E_{Df}$), a chemical activation energy was observed between CO_2_ and LDH-Ca_2_Al ($∆E_{Dc}\approx 5.2\;\mathrm{KJ}/\mathrm{mol}$) in the Δ*E* of the FKM/LDH-Ca_2_Al nanocomposites. Therefore, it has a significantly lower diffusion coefficient than that of FKM/GO/LDH-Ca_2_Al. The permeability coefficient of the composites is a combination of the diffusion coefficient and solubility (Q=DS) [5,48,62,63]. As shown in Figure 9c, the solubility of the nanocomposites substantially contributes to the permeability coefficient at 60–80 °C, resulting in the low permeability coefficient of FKM/GO/LDH-Ca_2_Al. By contrast, the diffusion coefficient of the nanocomposites substantially contributes to the permeability coefficient at 80–100 °C. Therefore, the permeability coefficient of the FKM/LDH-Ca_2_Al nanocomposites is lower than that of FKM/GO/LDH-Ca_2_Al.

### 3.8. Mechanical Properties of FKM/LDH-Ca_2_Al and FKM/GO/LDH-Ca_2_Al Nanocomposites

**Figure 10:**
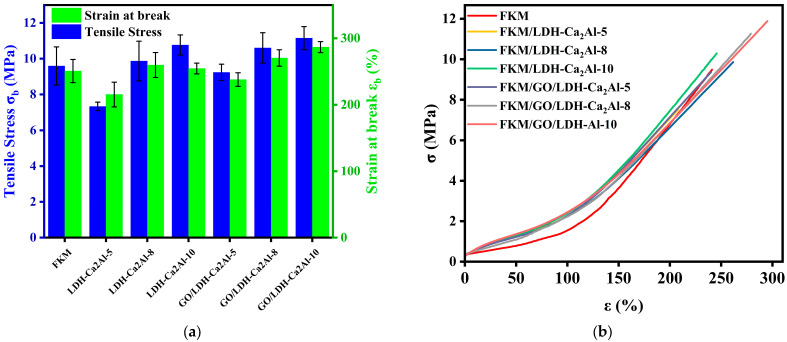
(**a**) Fracture strength (*σ_b_*) and strain (*ε_b_*). (**b**) stress–strain curves of FKM, FKM/LDH-Ca_2_Al, and FKM/GO/LDH-Ca_2_Al nanocomposites.

The effect of LDH-Ca_2_Al and GO/LDH-Ca_2_Al on the mechanical properties of fluoroelastomers was quantified by determining the key mechanical parameters of the nanocomposites with different filler contents (5, 8, and 10 phr), including 100% modulus (*E100*), Shore A hardness, *σ_b_*, and *ε_b_* (Figure 10 and Table 5). The mechanical properties of the nanocomposites were significantly improved compared with those of FKM, and interestingly, the *ε_b_* of the nanocomposites gradually increased with the content of nanofillers, in which the largest *ε_b_* (*ε_b_* > 287%, *σ_b_* > 11.1 MPa) value was obtained for the 10 phr filled FKM/GO/LDH-Ca_2_Al nanocomposites. This finding can be attributed to the orientation arrangement of the nanofillers with two-dimensional layered structures in the complexes when subjected to force, leading to the stable transfer of stress inside the material [9]. In addition, FKM/GO/LDH-Ca_2_Al nanocomposites exhibit superior mechanical properties to FKM/LDH-Ca_2_Al nanocomposites, in which *σ_b_*, and *ε_b_* increased by approximately 25% and 12% (Table 5) because of the strong interfacial interaction between GO/LDH-Ca_2_Al and the polymer matrix, which promotes the efficient transfer of stress from the polymer matrix to GO/LDH-Ca_2_Al, consistent with the results described by the phenomenological interaction parameters (Table 3) [19].

## 4. Conclusions

In this paper, the CO_2_ gas barrier and mechanical properties of nanocomposites filled with Ca/Al layered hydroxide modified by GO (GO/LDH-Ca_2_Al) were investigated. The GO/LDH-Ca_2_Al nanocomposite filler was prepared by depositing LDH-Ca_2_Al on the surface of alkalized graphene oxide (Al-GO). This nanocomposite filler has good thermal stability compared with the pristine LDH-Ca_2_Al, and the temperature of the stripped interlayer water increased by nearly 43 °C. At the same time, its CO_2_-resistant stability was significantly improved, and almost no carbonate generation occurred at 80 °C and 4.3 MPa pure carbon dioxide environment, resulting in low CO_2_ reactivity. The barrier mechanism of modified and unmodified LDH-Ca_2_Al-filled nanocomposites to carbon dioxide was also proposed, and the diffusion activation energy and solubility activation energy of the nanocomposites consisted of three components: (1) $∆H_{S0}$ and $∆E_{D0}$, which were determined by the nature of the matrix; (2) $∆H_{sf}$ and $∆E_{Df}$ due to packing effect; and (3) the compound effect $∆H_{sc}$ and reaction activation energy $∆E_{Dc}$. In the nanocomposites, the multilayer stacked LDH-Ca_2_Al acts as a CO_2_ gas barrier to form a distorted diffusion path, and this effect induces an activation energy of $∆E_{D0}$. Considering the pro-CO_2_ multilayer structure and strong chemical reactivity of LDH-Ca_2_Al, it provides the opportunity for CO_2_ intercalation and reaction, resulting in the reaction activation energy of $∆E_{Dc}$. These two factors led to an increase in the diffusion activation energy of FKM/LDH-Ca_2_Al nanocomposites by approximately 42.5%. The solubility activation energy of FKM/GO/LDH-Ca_2_Al nanocomposites increased by approximately 3.8 KJ/mol compared with that of FKM/LDH-Ca_2_Al nanocomposites, which was caused by the compound effect of GO and LDH-Ca_2_Al. This phenomenon explains the existence of strong interfacial interactions between GO/LDH-Ca_2_Al and the polymer matrix, thus reducing the CO_2_ adsorption effect on the nanofiller surface. The permeability coefficients of the nanocomposites to CO_2_ gradually decreased, and the mechanical properties (Shore A, *σ_b_*, and *ε_b_*) were significantly improved with the increase in nanofiller content. The best CO_2_ permeation resistance and mechanical properties were obtained for the FKM/GO/LDH-Ca_2_Al nanocomposites due to the strong interfacial interactions between GO/LDH-Ca_2_Al and FKM.

## Figures and Tables

**Table 1:** FKM nanocomposites formulation.

Samples	FKM 246	LUPEROX 101XL-50	TAIC	LDH-Ca_2_Al ^a^	GO/LDH-Ca_2_Al ^a^
FKM	100	5	5		
FKM/LDH-Ca_2_Al-5	100	5	5	5	5
FKM/LDH-Ca_2_Al-8	100	5	5	8	
FKM/LDH-Ca_2_Al-10	100	5	5	10	
FKM/GO/LDH-Ca_2_Al-5	100	5	5		5
FKM/GO/LDH-Ca_2_Al-8	100	5	5		8
FKM/GO/LDH-Ca_2_Al-10	100	5	5		10

^a^ The densities of the materials FKM 246, LDH-Ca_2_Al, and GO/LDH-Ca_2_Al are 1.86, 1.65, and 2.01 g/cm^3^, respectively.

**Table 2:** Contents of CaCO_3_ in LDH-Ca_2_Al, GO/LDH-Ca_2_Al, S-LDH-Ca_2_Al, and S-GO/LDH-Ca_2_Al ^a^.

Sample	Content (CaCO_3_) (%)
LDH-Ca_2_Al	0.23
GO/LDH-Ca_2_Al	5.97
S-LDH-Ca_2_Al	59.94
S-GO/LDH-Ca_2_Al	2.37

^a^ XRD data of LDH-Ca_2_Al, GO/LDH-Ca_2_Al, S-LDH-Ca_2_Al, and S-GO/LDH-Ca_2_Al were obtained via fine-tuning by using FullProf 2023.2 software.

**Table 3:** Phenomenological interaction parameter (B), $tan\;\delta_{m}$, tan δ, *T_g_*, and φ of FKM, FKM/LDH-Ca_2_Al-8, and FKM/GO/LDH-Ca_2_Al-8.

Sample	$tan\delta_{m}$	tanδ	*φ* (%)	B	*Tg* (°C)
FKM	1.480	-	-	-	3.3
FKM/LDH-Ca_2_Al-8	-	1.396	8.254	0.487	4.9
FKM/GO/LDH-Ca_2_Al-8	-	1.404	6.88	0.528	5.6

**Table 4:** Activation energy of D and S of FKM, FKM/LDH-Ca_2_Al-8, and FKM/GO/LDH-Ca_2_Al-8.

Sample	Activation Energy of D(ΔE) ^a^				Activation Energy of S(ΔH) ^a^			
	$D_{0}$ ^b^	$∆E_{D0}$	$∆E_{Df}$	$∆E_{Dc}$	$S_{0}$ ^c^	$∆H_{S0}$	$∆H_{sf}$	$∆H_{sc}$
FKM	2.6 × 10^−2^	−52.8	-	-	1.8 × 10^−11^	44.5	-	-
FKM/LDH-Ca_2_Al-8	1.0 × 10^−6^	−25.2	22.4	5.2	2.2 × 10^−6^	44.5	−32.2	-
FKM/GO/LDH-Ca_2_Al-8	7.7 × 10^−6^	−30.4	22.4	-	4.7 × 10^−7^	44.5	−32.2	3.8

^a^ the unit of activation energy is KJ/mol. ^b^ $D_{0}$ and ^c^ $S_{0}$ are the dimensionless constants.

**Table 5:** Mechanical properties of FKM, FKM/LDH-Ca_2_Al, and FKM/GO/LDH-Ca_2_Al complexes.

Sample	100% Modulus, *E100*(MPa)	Tensile Strength, *σ_b_* (MPa)	Strain at Break, *ε_b_* (%)	Shore A Hardness
FKM	1.5 ± 0.1	9.6 ± 1.1	251 ± 17	50 ± 1
FKM/LDH-Ca_2_Al-5	2.1 ± 0.2	7.3 ± 0.2	215 ± 18	55 ± 1
FKM/LDH-Ca_2_Al-8	2.3 ± 0.08	9.9 ± 1.1	260 ± 19	56 ± 1
FKM/LDH-Ca_2_Al-10	2.4 ± 0.03	10.8 ± 0.6	254 ± 8	58 ± 1
FKM/GO/LDH-Ca_2_Al-5	2.3 ± 0.01	9.2 ± 0.5	238 ± 10	55 ± 1
FKM/GO/LDH-Ca_2_Al-8	2.2 ± 0.03	10.6 ± 0.8	270 ± 12	56 ± 1
FKM/GO/LDH-Ca_2_Al-10	2.4 ± 0.08	11.1 ± 0.6	287 ± 8	57 ± 1

## Data Availability

The data presented in this study are available on request from the corresponding author.

## Supplementary Materials

The following supporting information can be downloaded at https://www.mdpi.com/article/10.3390/polym15204151/s1. Figure S1: SEM images of GO; Figure S2: TGA-DTG curves of GO.
