# Supplementary material for: Effect of Graphene Oxide-Modified CaAl-Layered Double Hydroxides on the Carbon Dioxide Permeation Properties of Fluoroelastomers
Source: Polymers (Basel). 2023 Oct 19;15(20):4151. doi: 10.3390/polym15204151 (PMC10610964; doi:10.3390/polym15204151)
Supplement: Supplementary file 1 [file polymers-15-04151-s001.zip › polymers-2634535-supplementary.pdf]

Supplementary File

Effect of Graphene Oxide-Modified CaAl-Layered Double Hydroxides on the Carbon Dioxide Permeation Properties of Fluoroelastomers

Chuanbo Cong *, Daigang Peng, Qingkun Liu, Mingyang Yuan, Xiaoyu Meng and Qiong Zhou

New Energy and Material College, China University of Petroleum, Beijing 102249, China; pengdg_123@163.com (D.P.); liuqingkun1992@163.com (Q.L.); yuanmy.apply@gmail.com (M.Y.); xymeng800418@sohu.com (X.M.); zhouqiong_cn@163.com (Q.Z.)
* Correspondence: congcb@cup.edu.com

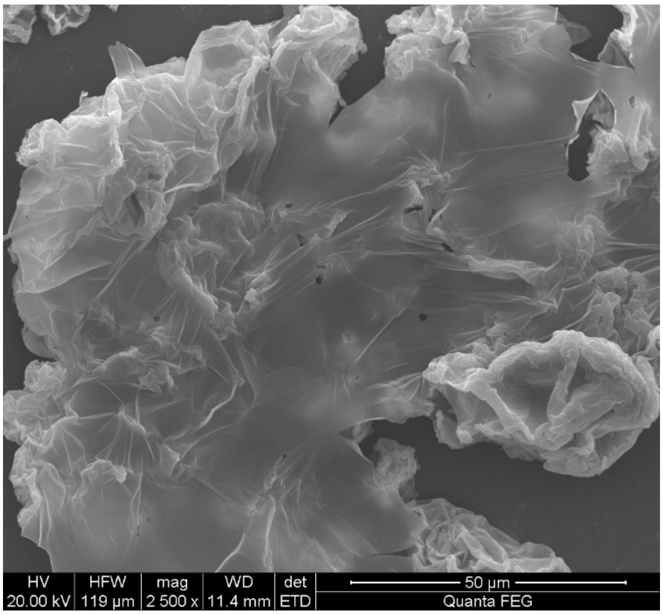

Figure S1. SEM images of GO.

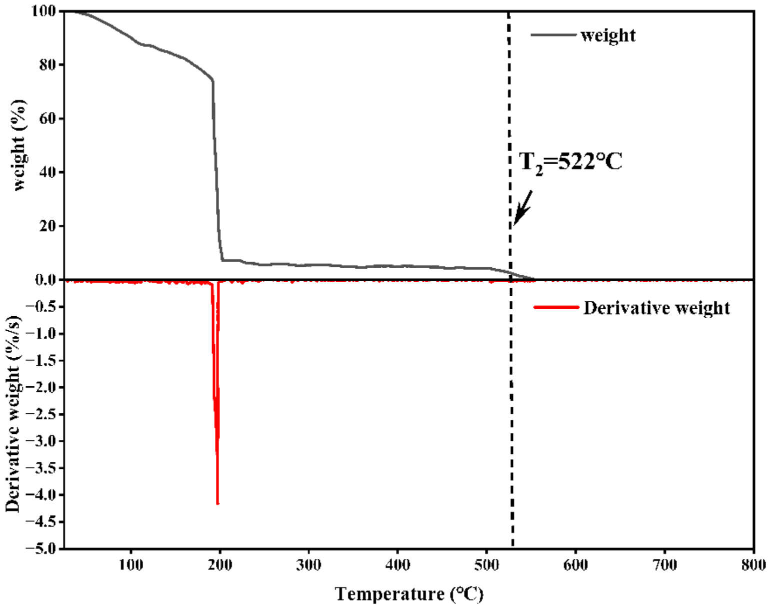

Figure S2. TGA-DTG curves of GO.
